# Three inflammation‐related genes could predict risk in prognosis and metastasis of patients with breast cancer

**DOI:** 10.1002/cam4.1962

**Published:** 2019-01-11

**Authors:** Shuangtao Zhao, Wenzhi Shen, Renle Du, Xiaohe Luo, Jiangyong Yu, Wei Zhou, Xiaoli Dong, Ruifang Gao, Chaobin Wang, Houpu Yang, Shu Wang

**Affiliations:** ^1^ Breast Disease Center, Peking University People’s Hospital Peking University Beijing China; ^2^ Department of Radiation Oncology, National Cancer Center/Cancer Hospital Chinese Academy of Medical Sciences and Peking Union Medical College Beijing China; ^3^ Department of Pathology and Institute of Precision Medicine Jining Medical University Jining China; ^4^ The School of Medicine Nankai University Tianjin China

**Keywords:** breast cancer, CYCS, prognosis, TBX21, TGIF2

## Abstract

**Background:**

Current predictive model is not developed by inflammation‐related genes to evaluate clinical outcome of breast cancer patients.

**Methods:**

With mRNA expression profiling, we identified 3 mRNAs with significant expression between 15 normal samples and 669 breast cancer patients. Using 7 cell lines and 150 paraffin‐embedded specimens, we verified the expression pattern by bio‐experiments. Then, we constructed a three‐mRNA model by Cox regression method and approved its predictive accuracy in both training set (n = 1095) and 4 testing sets (n = 703).

**Results:**

We developed a three‐mRNA (*TBX21*, *TGIF2,* and *CYCS*) model to stratify patients into high‐ and low‐risk subgroup with significantly different prognosis. In training set, 5‐year OS rate was 84.5% (78.8%‐90.5%) vs 73.1% (65.9%‐81.2%) for the low‐ and high‐risk group (HR = 1.573 (1.090‐2.271); *P = *0.016). The predictive value was similar in four independent testing sets (HR>1.600; *P < *0.05). This model could assess survival independently with better predictive power compared with single clinicopathological risk factors and any of the three mRNAs. Patients with both low‐risk values and any poor prognostic factors had more favorable survival from nonmetastatic status (HR = 1.740 (1.028‐2.945), *P* = 0.039). We established two nomograms for clinical application that integrated this model and another three significant risk factors to forecast survival rates precisely in patients with or without metastasis.

**Conclusions:**

This model is a dependable tool to predict the disease recurrence precisely and could improve the predictive accuracy of survival probability for breast cancer patients with or without metastasis.

## 1 INTRODUCTION

Breast cancer is reported as the most widespread tumor in women.^1^, ^2^ It is universally accepted that the adjuvant or neoadjuvant systemic therapy substantially improves the survival probability in patients with breast cancer.^3^, ^4^, ^5^, ^6^ Traditionally, the clinicopathological risk features such as age,^7^ tumor size,^8^ histologic type,^9^ status of axillary lymph nodes,^10^ and hormone‐receptor^11^, ^12^ could divide patients into high‐ and low‐risk subgroup, but they have limited predictive power. Then, many biomarkers are explored for predicting the prognosis of breast cancer, but they could not still satisfy clinical practice.^13^, ^14^, ^15^ Thus, it is urgently to investigate some new biomarkers to add the diagnostic power to the current predictive system.

Previous studies showed that the inflammatory microenvironment as the seventh hallmark of cancer could be activated to promote tumor process.^16^ Zhao et al discovered that three inflammatory genes (*IL‐6*, *IL‐1A,* and *CSF3*) could predict prognosis of patients with diffuse large B‐cell lymphoma,^17^ and the inflammation‐related gene *TBX21* could evaluate the survival of patients and increase cancer stemness via the TBX21‐IL‐4 pathway in lung adenocarcinoma.^18^ Loza et al summarized that the pathway comprised of inflammation genes could define genetic risk factors for cancers.^19^ Currently, there is no data regarding a reliable model including some inflammatory genes to predict prognosis of patients with breast cancer.

To address this knowledge gap, we develop a three‐mRNA model including three inflammation‐related genes with the Cox regression method to evaluate prognosis independently and predict survival probability precisely in breast cancer patients with or without metastasis from The Cancer Genome Atlas (TCGA). Then, we validated the generalization ability of this model in another four independent cohorts from Gene Expression Omnibus (GEO) datasets. And we also compared its diagnostic power to single mRNAs and clinicopathological risk factors.

## 2 METHODS

### 2.1 Patients and samples

The clinical materials and mRNA data were collected from a research team (Cat. #BR1504a, Shanxi, Alenabio company, China), The Cancer Genome Atlas (TCGA) (https://cancergenome.nih.gov), and GEO datasets (https://www.ncbi.nlm.nih.gov/gds). After the removal of missing values, a total of 1938 breast cancer patients and 25 normal samples were applied into in this study, including 140 patients and 10 normal samples from Alenabio company, 1095 patients from TCGA, 249 patients from GSE21653, 237 patients from GSE4922, 138 patients from GSE22226, 79 patients from GSE58812, and 15 normal controls from GSE8977 separately. The normalization of expression values in studied genes was completed with a house‐keeping gene GAPDH. The formalin‐fixed paraffin‐embedded (FFPE) specimens of the 140 patients and 10 normal controls were collected between 2003 and 2006 and pathologically confirmed by the pathologist in the Nankai University.

### 2.2 Screening process of the significant genes

A total of 156 candidate genes were screened from these 1027 inflammation‐related genes by a medium throughput RNAi screen platform.^20^ And then, 14 significant genes were collected from these 156 primary screening genes in breast cancers between with and without metastasis (*P* < 0.15, *t* test method, Table S1). Finally, the three significant genes (TBX21, TGIF2, and CYCS) were obtained from above genes among the metastatic, relapse and tumorigenesis group when *P* < 0.15 in 2 of 3 compared groups with *t* test method, which were on behalf of the clinical characteristics of the cancer stemness.

### 2.3 Bio‐experiment methods

The biology experiment methods applied into this study including cell culture, real‐time PCR, Western blotting, and immunohistochemistry were similar with the research methods as described previously.^18^, ^20^ Primers used for the experiments are summarized in Table S2.

### 2.4 Functional enrichment analysis

The bioinformatical analysis methods were analogous with our research methods as described previously.^18^ Meanwhile, we applied the STRING tool (https://string-db.org/) to perform the functional protein association networks.

### 2.5 Statistical analysis

We defined a median as the cutoff value in the expression of the studied genes in each set. And Pearson's chi‐square test method was applied to analyze the statistical significance in the basic characteristics.^21^ The *t* test was performed to distinguish the distributive difference of the studied genes’ expression between cancers and healthy samples or between the high‐ and low‐risk groups. The correlation analysis between two genes in this signature was measured with Spearman correlation analysis in the TCGA set. In the overall survival (OS) analyses, Random Survival Forest (RSF) algorithm and Cox proportional hazards regression analysis were used to screen the optimal method to construct the prognostic model. Next, the Kaplan‐Meier method was used to analyze the relationship between factors and overall survival, and the log‐rank test to compare survival curves. The Cox regression model was selected to complete the multivariate survival analysis and data stratification analysis for exploring the independency of this signature model.^22^ Hazard ratios (HRs) and 95% confidence intervals (CIs) were computed in each dataset. The prognostic performance at 3, 5, and 10 years was evaluated by time‐dependent ROC curves. The Cox regression coefficients were to generate nomograms, and bootstrap cross‐validation method was selected to prove their predictive accuracy. Meanwhile, we used calibration to assess whether the actual results approximate the predicted outcomes for each nomogram. The nomogram and calibration plots were performed with *rms* R package, and the other statistical tests were conducted with R software (version 3.4.4). *P* < 0.05 was defined as the significant threshold in statistical results.

## 3 RESULTS

### 3.1 Identification and derivation of a three‐mRNA model in predicting prognosis for patients with breast cancer

**Figure 1:**
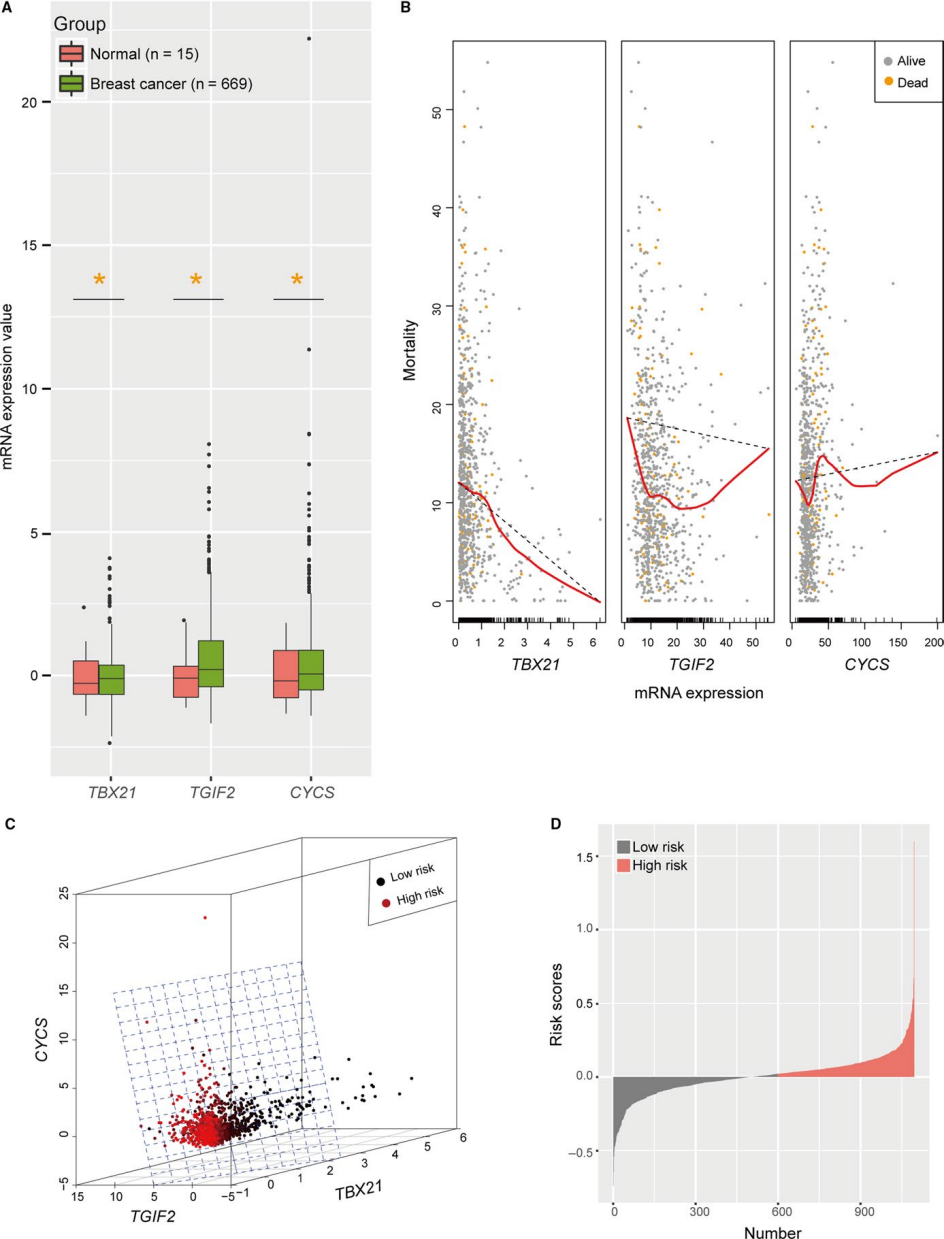
The model construction. A, Different expression of three significant mRNAs between breast cancers and normal controls (*P < 0.05, *t* test method). B, Scatter plot shows three mRNA expression values to dead as a first event for those in whom this occurred (yellow dots), and alive for all other patients (gray dots). Shafts on the x‐axis represent breast cancer patients. Red spline and dotted line represent the fitted trend and the overall trend of mortality for patients, respectively. C, Three‐dimensional stereogram shows three‐mRNA‐based classifier stratifies patients into high (red dots)‐ and low (black dots)‐risk group with median as a cutoff value. D, Histogram shows three‐mRNA‐based classifier divides patients into high (red color)‐ and low (black color)‐risk group with median of predictive scores as a cutoff value

To develop a predictive model, we compared the expression values of 1027 inflammation‐related genes^18^, ^19^ and obtained three significant mRNAs (*TBX21*, *TGIF2,* and *CYCS*) between 669 breast cancer patients from TCGA and 15 normal controls from GSE8977 (*P* < 0.05; Figure 1A). In line with above, the expression patterns of these three genes were verified in 7 cell lines (Figure S1A‐C) and 150 tissues from normal (n = 10) and cancer (n = 140) samples (Figure S1D‐F). Then, we discovered that the mortality was negatively correlated with the expression value of *TBX21 *and *TGIF2*, but positively with *CYCS* in training set (n = 1095, Figure 1B). With no multicollinearity (Spearman's r ≤ 0.15; Figure S2A), these genes were necessary for this new model by RSF analysis (depth ≥ 0.5; Figure S2B). Considering a balance between the discriminant error rate and the number of mRNAs (Figure S2C), we finally integrated three mRNAs into a predictive model with Cox regression algorithm: Risk scores = −0.082*TBX21‐ 0.05*TGIF2 + 0.069*CYCS. With a cutoff point defined by the median value^17^, ^18^, ^22^ (0.020, Figure 1C,D), patients with high‐risk scores (risk score>0.020) were classified into high‐risk group. In contrast, the patients with risk score ≥0.020 were divided into low‐risk group. The basal characteristics (Table 1) were not significantly different between the low‐ and high‐risk groups except for race (*P* = 0.014), tumor stage (*P* = 0.010), copy number aberration (CNA, *P* = 0.040), surgical margin status (*P* < 0.0001), and menopause status (*P* = 0.014).

**Table 1:** Baseline characteristics of patients by 3‐mRNA signature in TCGA dataset

	Number of patients	Low risk (%)	High risk (%)	*P*‐value
Gender				
Male	10	7 (70)	3 (30)	0.527
Female	990	558 (56)	432 (44)	
Age				
≤60	553	300 (54)	253 (46)	0.110
>60	447	265 (59)	182 (41)	
Race				
White	718	391 (54)	327 (46)	0.014
Nonwhite	187	83 (44)	104 (56)	
ER status				
Negative	237	123 (52)	114 (48)	0.073
Positive	804	470 (58)	334 (42)	
PR status				
Negative	342	201 (59)	141 (41)	0.424
Positive	698	392 (56)	306 (44)	
HER2 status				
Negative	557	321 (58)	236 (42)	0.223
Positive	355	190 (54)	165 (46)	
Tumor stage				
≤II	847	464 (55)	383 (45)	0.010
>II	153	101 (66)	52 (34)	
Lymph node stage				
0	471	271 (58)	200 (42)	0.533
≥1	529	294 (56)	235 (44)	
Metastasis status				
Yes	428	228 (53)	200 (47)	0.475
No	667	370 (55)	297 (45)	
CNA				
≤0.251	539	275 (51)	264 (49)	0.040
>0.251	538	308 (57)	230 (43)	
Surgical margin status				
Positive	102	87 (85)	15 (15)	<0.0001
Negative	844	468 (55)	376 (45)	
Mutation count				
≤30	489	269 (55)	220 (45)	0.252
>30	486	285 (59)	201 (41)	
Neoadjuvant therapy				
Yes	11	9 (82)	2 (18)	0.088
No	988	555 (56)	433 (44)	
Tumor location				
Left	523	298 (57)	225 (43)	0.749
Right	477	267 (56)	210 (44)	
Clinical stage				
≤II	761	419 (55)	342 (45)	0.088
>II	238	146 (61)	92 (39)	
Menopause status				
≤12 mo since LMP	280	179 (64)	101 (36)	0.014
>12 mo since LMP	646	357 (55)	289 (45)	
Neoplasm status				
Tumor‐free	772	421 (55)	351 (45)	0.893
With tumor	80	43 (54)	37 (46)	

TCGA, The Cancer Genome Atlas.

### 3.2 The three‐mRNA model predicted the prognosis effectively in a training set and 4 independent testing sets

**Figure 2:**
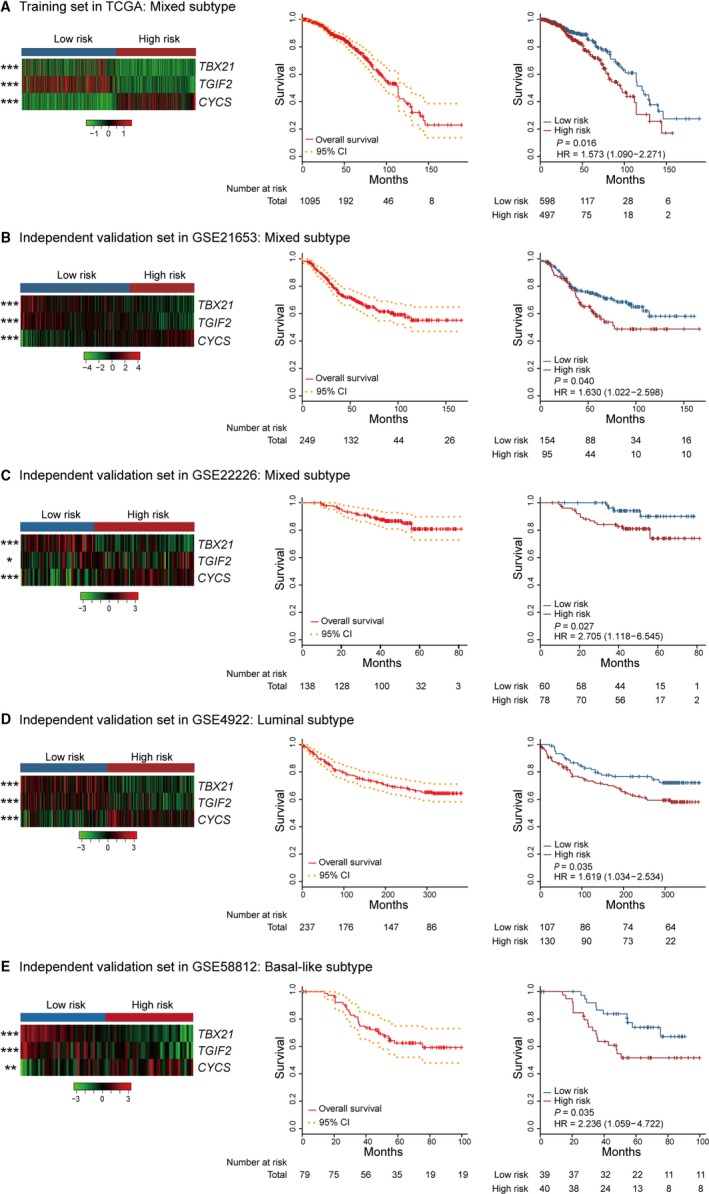
Risk stratification by the model, and overall survival analysis by Kaplan‐Meier method in the whole patients and between high‐ and low‐risk group. A, training cohort. B‐E, independent testing cohorts. CI, confidence interval; HR, hazard ratio

Applying this new model into clinical practice, we found that the mRNA expression of *TBX21* and *TGIF2* in breast cancer patients with high‐risk value was significantly lower than those with low‐risk value (*P < *0.0001), but inversely for *CYCS*. The 5‐year OS rates were 79.1% (74.4%‐84.0%) for all patients, 84.5% (78.8%‐90.5%) in the low‐risk group (n = 598), and 73.1% (65.9%‐81.2%) in high‐risk group (n = 497), respectively (HR = 1.573 (1.090‐2.271), *P* = 0.016; Figure 2A). These results pointed out that this model could discriminate breast cancer patients with high or low risk of survival.

To validate the generalization of this new model, a total of 703 breast cancer patients were classified into low and high subgroups with the same cutoff point in another 4 independent testing sets (GSE21653, GSE4922, GSE22226, and GSE58812). The 5‐year OS rates were 67.7% (61.6%‐74.3%) for all patients (n = 249), 73.1% (66.0%‐81.0%) in low‐risk group (n = 154) and 58.2% (47.9%‐70.7%) in high‐risk group (n = 95), respectively, in GSE21653 (HR = 1.630 (1.022‐2.598), *P* = 0.040; Figure 2B). For the other three testing sets, the 5‐year OS rates were analogous with the result above in all patients, high‐ and low‐risk group (Figure 2C‐E). Therefore, these results indicated that the three‐mRNA model was powerful to identify the prognosis of breast cancer patients with luminal, Her2+, or basal‐like subtype.

### 3.3 Prognostic prediction by the three‐mRNA model was independent of clinicopathological factors

**Figure 3:**
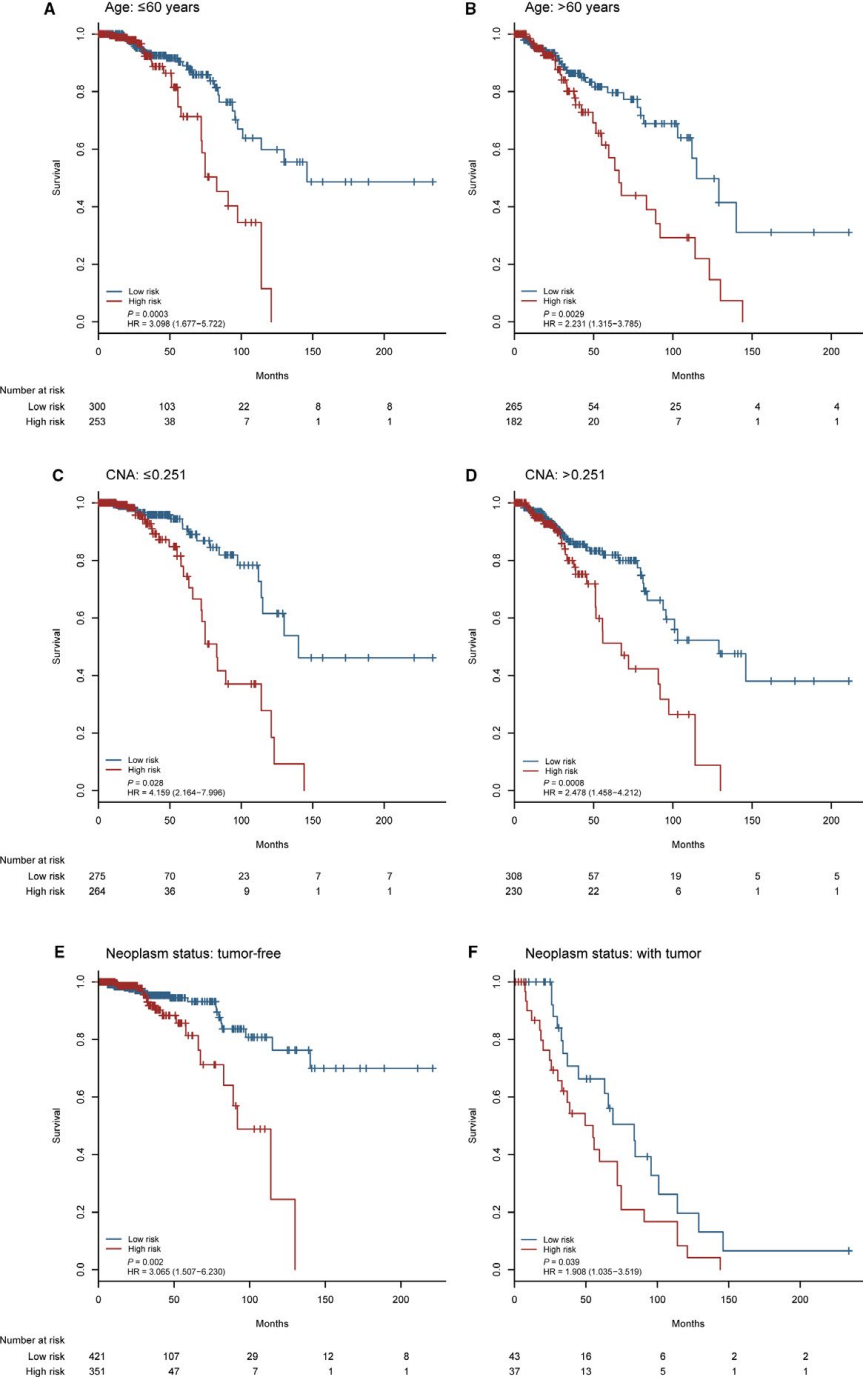
Kaplan‐Meier survival analysis for all patients with breast cancer in TCGA set according to the model stratified by significantly clinicopathological risk factors. A‐B, Patients’ age. C‐D, CNA. E‐F, Neoplasm status. *P*‐values were calculated by the log‐rank test. CNA: copy number aberration; HR: hazard ratio

To assess the independence of this model in predicting prognosis, we performed multivariate Cox regression analysis and discovered this model significantly associated with survival when adjusted for the other 14 clinical variables in TCGA set, as well as age, CNA, and neoplasm status (HR = 1.843 (1.094‐3.104), *P* = 0.022; Table 2). Next, data stratification analysis was completed between age, CNA, or neoplasm status. All patients were divided into young group (≤ 60 years, n = 553) and older group (>60 years, n = 447). As shown in Figure 3A, the cutoff value of the model could subclassify young patients into high‐ and low‐risk group with significant prognosis (HR = 3.098 (1.677‐5.722), *P* = 0.0003). The 5‐year OS rates of patients with high‐risk scores were 71.3% (95% CI: 58.5‐86.9), which was also significantly decreased compared with patients with low‐risk scores whose corresponding proportions were 89.0% (83.6‐94.7). For the older group, the model exposed the comparable prognostic power (*P* = 0.0029; Figure 3B). Subsequently, the model was further measured in patients with different CNA or neoplasm status. The patients from each subgroup were subclassified into high‐ and low‐risk group with dramatically different prognosis (*P* < 0.05). The 5‐year OS rates of patients with high‐risk scores were notably worse than those with low‐risk scores (74.4% vs 90.9% in less CNA group (≤ 0.251; *P* = 0.028; Figure 3C) and 81.4% vs 93.2% in tumor‐free group (*P* = 0.002; Figure 3E); median survival time (months): 67.4 vs 129.0 in more CNA group (>0.251; *P* = 0.0008; Figure 3D) and 55.0 vs 83.8 in with tumor group (*P* = 0.039; Figure 3F)). These results demonstrated that the prognostic power of the new model is independent of other clinicopathological characteristics for breast cancer patients.

**Table 2:** Univariate and multivariate Cox regression analysis of the three‐mRNA‐based classifier in TCGA set

Factors	Univariate analysis		Multivariate analysis	
	HR (95% CI)	*P*‐value	HR (95% CI)	*P*‐value
Three‐mRNA‐based classifier (High risk/Low risk)	2.438 (1.684‐3.532)	0.000	1.843 (1.094‐3.104)	0.022
Gender (Male/Female)	0.049 (0.000‐792.724)	0.542		
Age (>60/≤60)	1.793 (1.248‐2.577)	0.002	2.872 (1.597‐5.167)	0.000
Race (White/Nonwhite)	1.086 (0.676‐1.746)	0.733		
ER status (Positive/Negative)	0.777 (0.516 ‐ 1.172)	0.229		
PR status (Positive/Negative)	0.692 (0.472 ‐ 1.014)	0.059		
HER2 status (Positive/Negative)	1.106 (0.676 ‐ 1.810)	0.687		
Tumor stage (>II/≤II)	1.187 (0.768‐1.835)	0.440		
Lymph node stage (≥1/0)	2.524 (1.687‐3.776)	0.000	1.161 (0.622‐2.167)	0.638
Metastasis status (Yes/No)	1.595 (1.109‐2.296)	0.012	1.503 (0.936‐2.415)	0.092
CNA (>0.251/≤0.251)	1.781 (1.224‐2.592)	0.003	2.043 (1.229‐3.396)	0.006
Surgical margin status (Positive/Negative)	1.357 (0.802‐2.296)	0.255		
Mutation count (>30/≤30)	1.118 (0.771‐1.621)	0.555		
Neoadjuvant therapy (Yes/No)	4.985 (1.560‐15.927)	0.007	3.033 (0.870‐10.571)	0.082
Tumor location (Right/Left)	0.747 (0.516‐1.080)	0.121		
Clinical stage (>II/≤II)	1.885 (1.284‐2.767)	0.001	1.201 (0.655‐2.203)	0.553
Menopause status (>12/≤12 mo since LMP)	1.812 (1.149‐2.859)	0.011	1.530 (0.784‐2.986)	0.213
Neoplasm status (With tumor/Tumor‐free)	6.814 (4.510‐10.295)	0.000	7.573 (4.465‐12.845)	0.000

TCGA, The Cancer Genome Atlas.

### 3.4 The three‐mRNA model had strongly diagnostic power in the prognostic prediction

**Figure 4:**
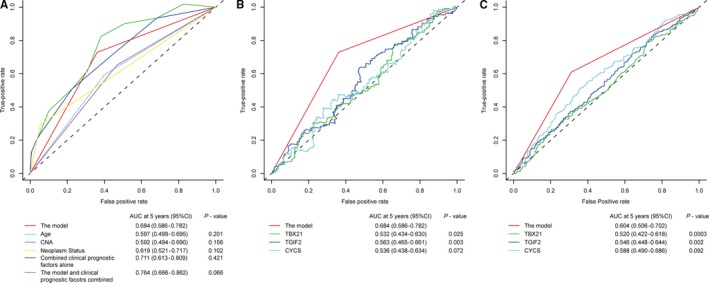
Time‐dependent ROC curves compare the prognostic accuracy of the model with clinicopathological risk factors and single mRNA in patients from the training set and all testing tests. (A) Comparison of the prognostic accuracy by the model (high vs low risk), Age (≤60 y vs >60 y), CNA (≤0.251 vs >0.251), neoplasm status (with tumor vs tumor‐free), combined clinical prognostic factors alone, or the classifier and clinicopathological prognostic factors combined. (B‐C) Comparisons of the prognostic accuracy by the model (high vs low risk), and *TBX21* (low vs high expression), *TGIF2* (low vs high expression), or *CYCS *(high vs low expression) in the training set (B) and the testing set (C). *P*‐values show the AUC at 5 y for the model vs the AUC at 5 y for other features. ROC: receiver operator characteristic; AUC: area under curve; CI: confidence interval; CNA: copy number aberration

To verify its strongly predictive power in prognosis, we calculated the area under the curve (AUC) value at 5 years with the time‐dependent ROC analysis among the significant clinical factors, three mRNAs, and this new model. We found that AUC value (0.684 (0.586‐0.782)) of this model was much higher than any single AUC value from the three clinical factors (0.597 (0.499‐0.695) for age, 0.592 (0.494‐0.690) for CNA, 0.619 (0.521‐0.717) for neoplasm status; Figure 4A) and the three mRNAs (0.532 (0.434‐0.630) for *TBX21*, 0.563 (0.465‐0.661) for *TGIF2*, 0.536 (0.438‐0.634) for *CYCS*; Figure 4B), which was validated in the testing set (n = 703) integrated by another 4 independent datasets (Figure 4C), although not all results reached statistically significant. These data indicated that the model was not only much higher prognostic accuracy than any other clinicopathological risk factors or single mRNA alone, but also added diagnostic power to clinicopathological prognostic features.

### 3.5 The three‐mRNA model improved the survival identification between metastatic and nonmetastatic breast cancers

**Figure 5:**
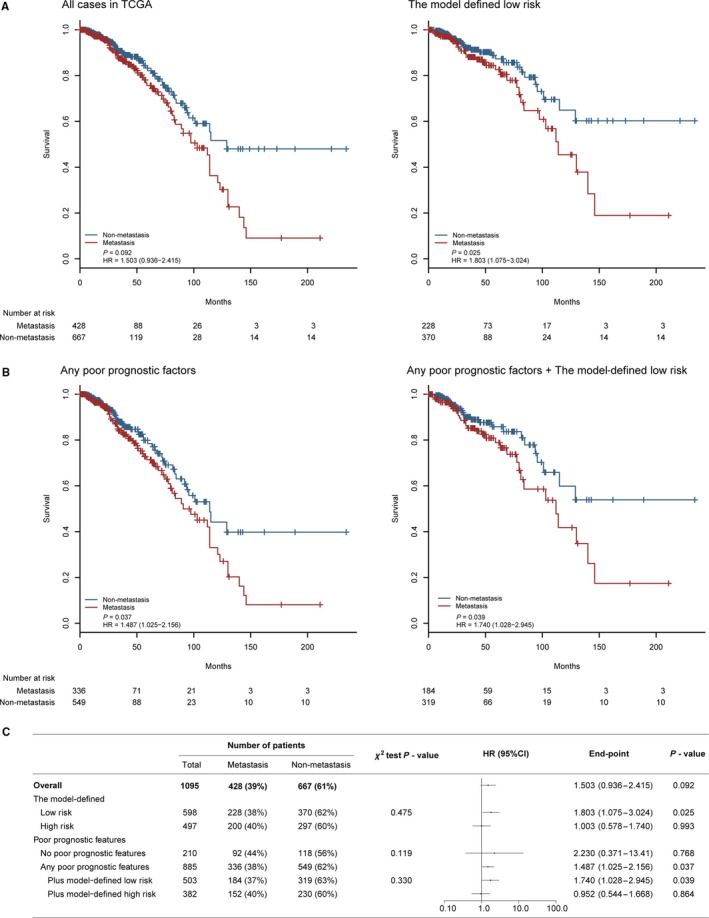
Effect of metastasis in different subgroups. A‐B, Kaplan‐Meier survival curves for patients in different subgroups stratified by the metastatic status. C, Effect of metastatic status on OS in different subgroups

To investigate whether the model was clinically associated with the metastasis, a special analysis of the three mRNAs was conducted between two groups with or without metastasis in TCGA set. As a result, we noted that the survival status was not identified between breast cancers with or without metastasis (HR = 1.503 (0.936‐2.415), *P* = 0.092; Figure 5A,C). Results from another subset analysis using our model showed that patients in the low‐risk group had a significantly favorable clinical outcome in nonmetastatic group compared with the metastatic one (HR = 1.803 (1.075‐3.024), *P = *0.025; Figure 5A,C), or in patients with any poor prognostic features (HR = 1.487 (1.025‐2.156), *P = *0.037; Figure 5B,C). Furthermore, patients with both low‐risk scores and any poor prognostic factors had a much better survival benefit from nonmetastatic status (HR = 1.740 (1.028‐2.945), *P = *0.039; Figure 5B,C). The results above indicated that the model could successfully improve the survival identification between breast cancer patients with or without metastasis.

### 3.6 The three‐mRNA model predicted the survival probability precisely by integrating other clinicopathological risk factors

**Figure 6:**
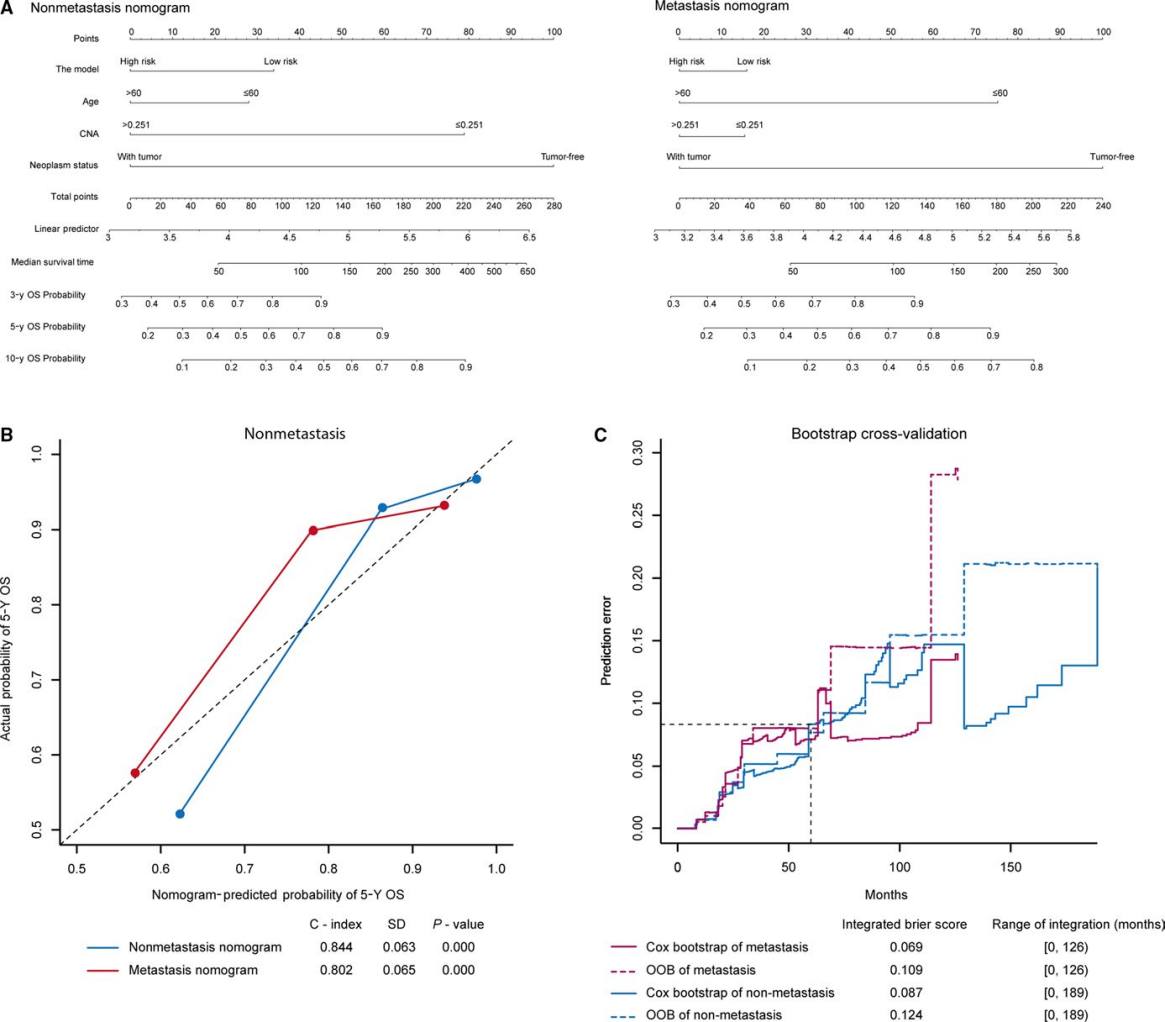
Nonmetastasis and metastasis nomograms to predict risk of cancer recurrence without or with metastasis in patients with breast cancer. A, Un‐metastasis and metastasis nomograms for predicting proportion of breast cancer patients with OS, either without (left) or with (right) metastasis status. B, Plots display the calibration of two models in terms of agreement between predicted and observed 5‐year survivals. The plot shows the model performance, relative to the 45‐degree line, which embodies perfect prediction. C, Validation of the predictive power of the nomograms’ model by bootstrap cross‐validation method. CNA, copy number aberration; OOB, out of bag; OS, overall survival; SD, standard deviation

To explore a quantitative method for calculating precise probability of cancer recurrence with or without metastasis, we developed two nomograms—metastasis and nonmetastasis—that integrated both the model and another three significant clinical risk factors (Figure 6A). Calibration plots displayed that C‐index of nonmetastasis nomogram (0.844 ± 0.063, *P* = 0.000) was similar with that of metastasis nomogram (0.802 ± 0.065, *P* = 0.000), which indicated that the nomograms worked well compared with an ideal model (Figure 6B). Further validation was performed when we applied bootstrap cross‐validation algorithm into the patients from TCGA set. The integrated Brier Score for Cox bootstrap of metastasis (0.069) was less than out of bag (OOB) of metastasis (0.109) at the range of integration (0, 126) months, which was similar with the result from nonmetastasis status (Figure 6C). Meanwhile, the AUC values of nonmetastatic and metastatic nomogram were 0.656 (0.552‐0.760) and 0.628 (0.528‐0.728), respectively (Figure S3). Generally, these nomograms could predict the survival probability accurately in patients with breast cancer as a practical clinical tool.

## 4 DISCUSSION

In the past few years, the study correlated with mRNAs in breast cancer progress was increased inch by inch.^23^, ^24^, ^25^ However, little has been reported for the inflammation‐related genes to predict breast cancer survival in a large dataset. Here, a three‐mRNA model was developed as a markedly predictor of survival in breast cancer patients with little overlap with the others. To reduce the predictive error rates, the random sampling and ensemble strategies were used in RSF algorithm and Cox regression method. And then, the measures of genes importance were performed by RSF algorithm to result in remarkable performance in factors screening. Next, a model was constructed with three mRNAs by Cox regression method. We espoused that the prognostic value could be substantially improved by integrating multiple biomarkers into a single model.^26^ However, it would be greater to have fewer genes as possible to produce the new model more competitive. Through the RSF analysis, the three‐mRNA signature was necessary to develop the final model. And the result validated it as the “less‐gene‐possible” combination could subclassify the breast cancer risk status effectively.

To identify survival of the three‐mRNA model of either metastatic or nonmetastatic breast cancers on principle of “less‐gene‐possible” combination, we select one published model including two genes (HOXB13 and IL17BR) reported by Ma et al^27^ to compare the predictive power of prognosis in breast cancer patients from the TCGA dataset. By using ROC analysis, we found that AUC value of the three‐mRNA model was higher than the two‐gene model in the metastatic group (0.630 vs 0.541, Figure S3A) and nonmetastatic group (0.650 vs 0.501, Figure S3B) of breast cancer patients. The data suggested that our three‐mRNA model has more strongly diagnostic power for predicting the clinical outcome in breast cancers.

To further verify the independence of this model in prediction, we assessed the correlation between this model and the basic clinical factors and identified three factors (age, CNA, and neoplasm status) as dramatically candidate predictive variables in training set. Then, we performed a multivariate Cox regression analysis and discovered this model as an independent factor. Especially the model could stratify patients with poor or favorable survival in the same age, CNA, or neoplasm status stratum, which demonstrated that the potential application of this model in adding diagnostic power to the combined clinicopathological factors. Because the positive metastasis status negatively influenced the prognosis of patients with breast cancer,^28^ the predictive value of this model discovered in TCGA set furtherly verify its remarkable association with metastasis. The nomograms developed by the model and another three significant risk factors produced a quantitative tool for predicting survival of patients. These results verified again that this model could divide the patients into high‐ and low‐risk group more effectively and precisely than the performance of any other single mRNA or clinical factor.

Interestingly, we discovered the important tradeoffs with respect to the application of the three‐mRNA model in breast cancer patients who were deemed to be at high or low risk of recurrence based on the clinical and pathological factors. Also, we could report the median survival time, 3/5/10‐year OS probability for these patients with or without metastasis by adding the three‐mRNA model into the nomogram. Overall, we could identify 40% and 60% in breast cancer patients with or without metastasis as especially short survival by evaluating individuals who had low‐ or high‐risk scores along with the basal clinical characteristics. This study gives us a big hint that we should pay enough attention to these patients in clinical treatment.

Importantly, it remains unknown whether this model has similar predictive power beyond molecular subtypes in breast cancer patients from different hospitals in China as this predictive model was derived from the TCGA set. Another limitation of this research is that the validity of this model should be further confirmed in the prospect cohorts, especially for the correlation with tumor size. In addition, the possible function of this signature in breast cancer could be deduced by performing functional enrichment analysis on GO terms and KEGG pathways (Figure S5). It is a plausible inference from the result that this signature might be involved in cell apoptotic process and differentiation, energy metabolism, and pathways in cancer. However, these discoveries should be verified by bio‐experimentation.

Generally, our study identified three mRNAs (*TBX21*, *TGIF2,* and *CYCS*) that were significantly altered between high‐ and low‐risk patients with breast cancer. The three‐mRNA model was independent and predicted the prognosis of patients robustly. Furthermore, this model could predict survival probability precisely in patients with or without metastasis. And it is the first predictive model developed by inflammation‐related mRNA signature to evaluate survival of patients with breast cancer.

## CONFLICT OF INTEREST

The authors declare that they have no competing interests.

## ETHICS APPROVAL AND CONSENT TO PARTICIPATE

The protocols in this study were reviewed and approved by the Ethical Committee and Institutional Review Board of Peking University People's Hospital, and written informed consents were collected from each patient included in this research. And this study did not involve the use of any animal tissue.

Consent for publication: All participants give their permission to publish this study.

## AVAILABILITY OF DATA AND MATERIALS

The datasets during and/or analyzed during the current study are available from the corresponding author on reasonable request.
