# Supplementary figures and images for: Three inflammation‐related genes could predict risk in prognosis and metastasis of patients with breast cancer
Source: Cancer Med. 2019 Jan 11;8(2):593–605. doi: 10.1002/cam4.1962 (PMC6382731; doi:10.1002/cam4.1962)

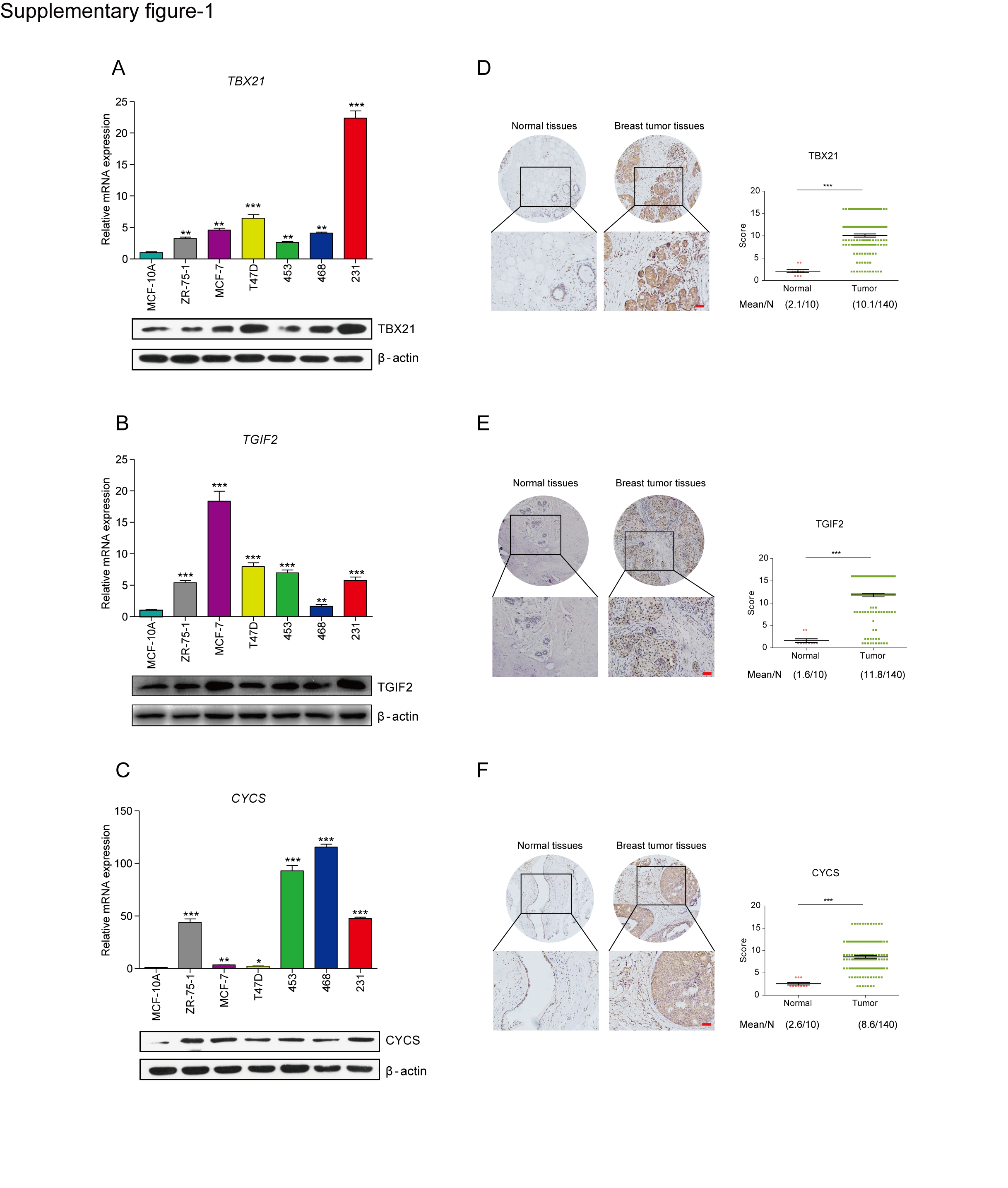

Supplement: Supplementary file 1 [file CAM4-8-593-s001.tif]

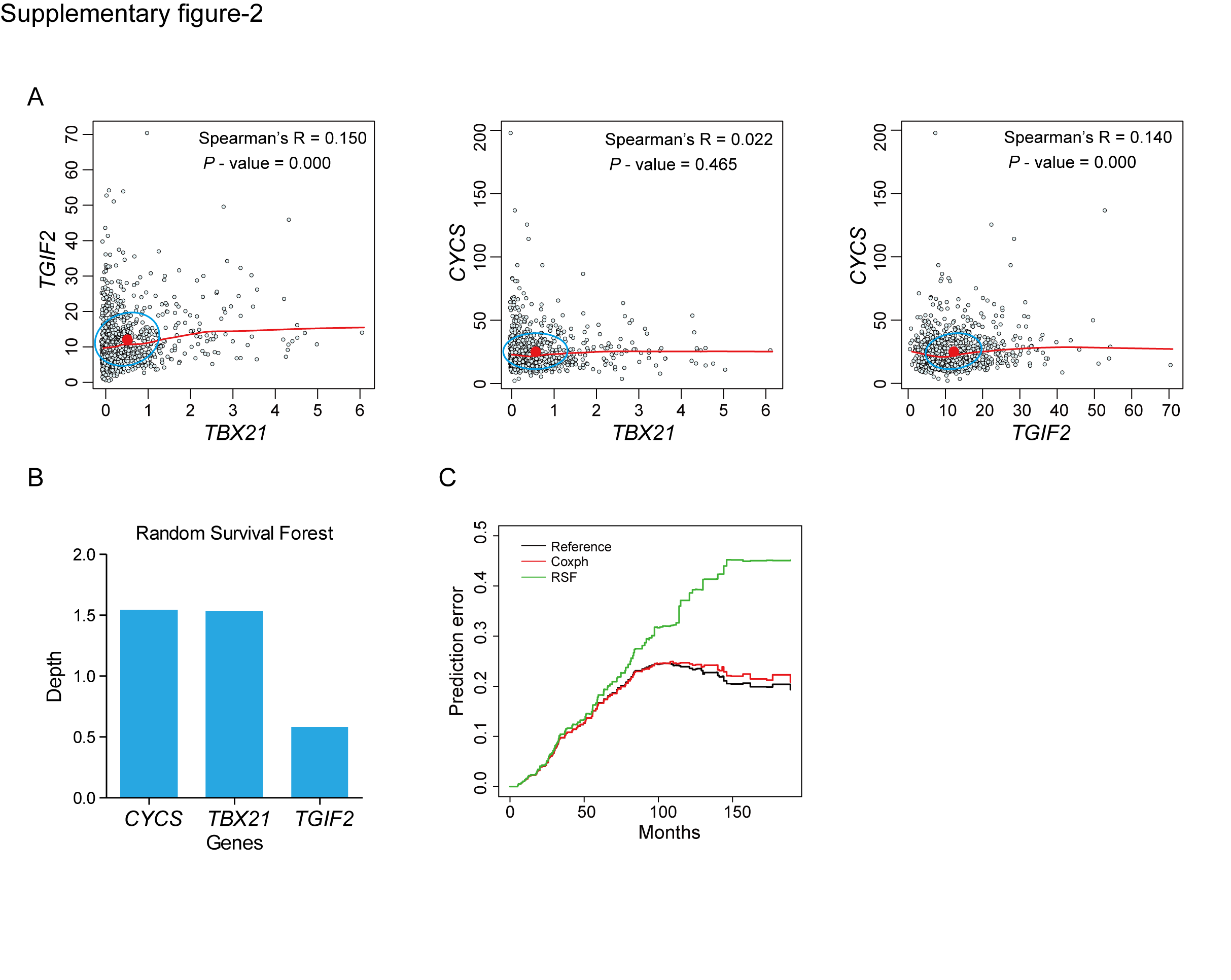

Supplement: Supplementary file 2 [file CAM4-8-593-s002.tif]

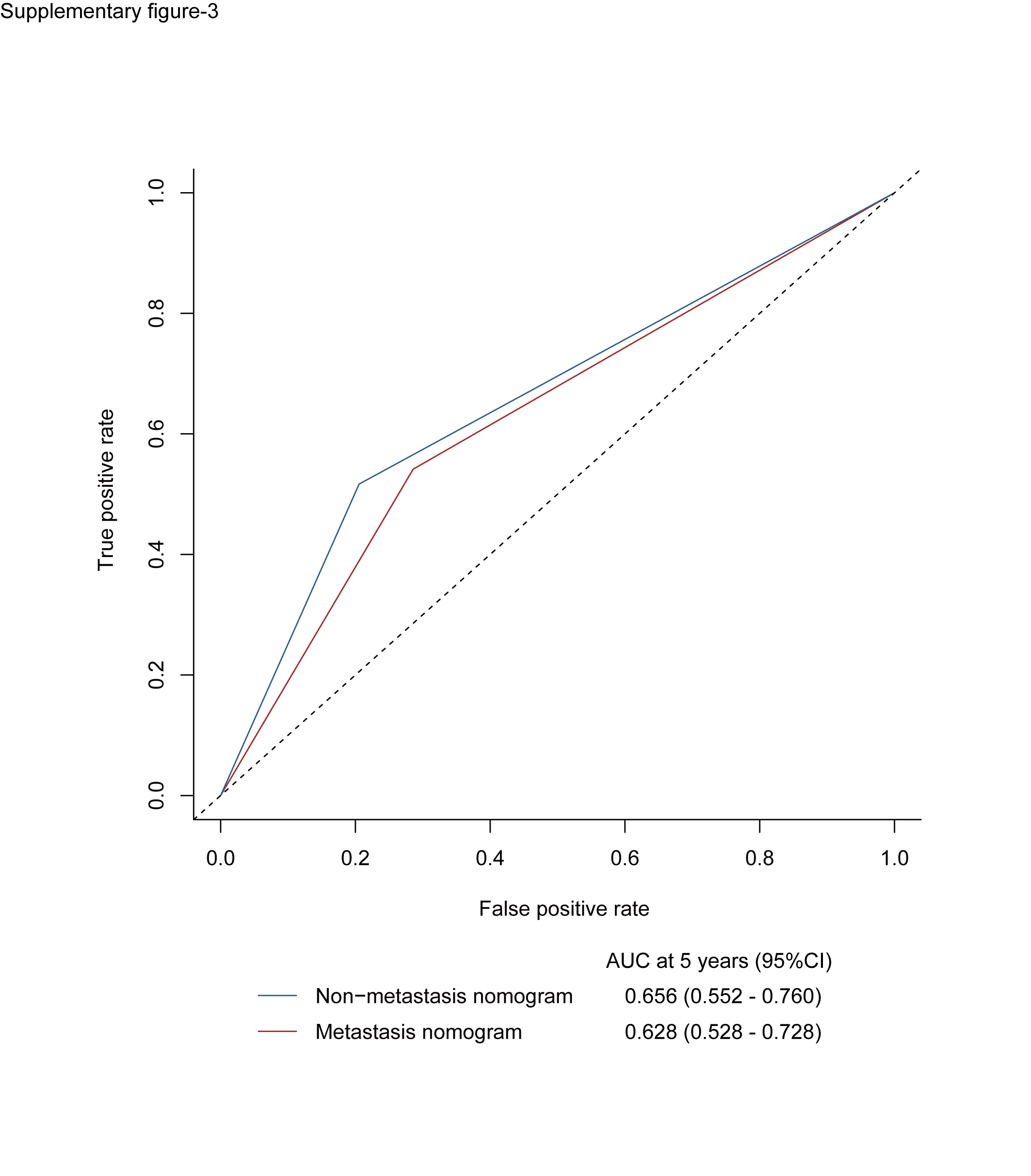

Supplement: Supplementary file 3 [file CAM4-8-593-s003.tif]

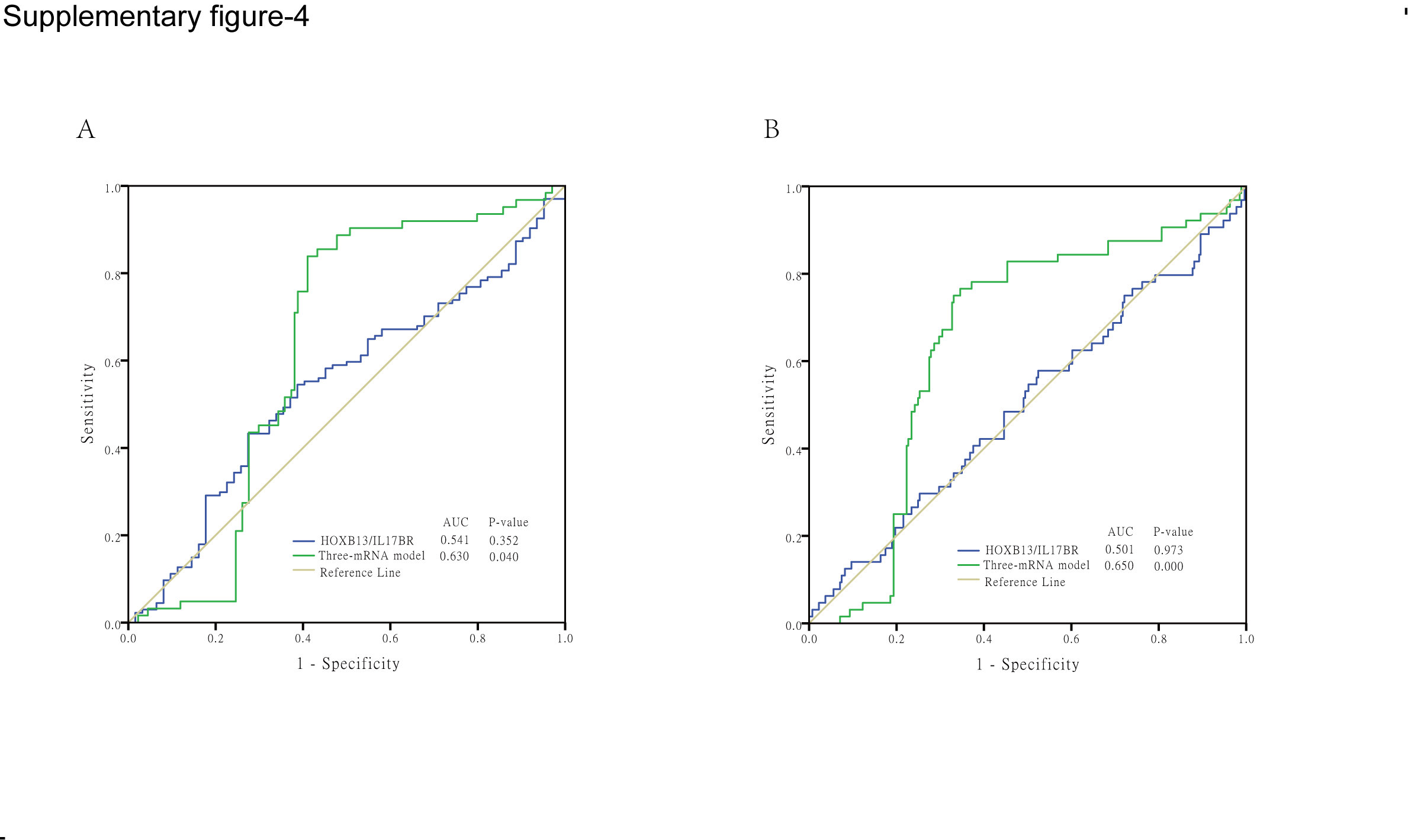

Supplement: Supplementary file 4 [file CAM4-8-593-s004.tif]

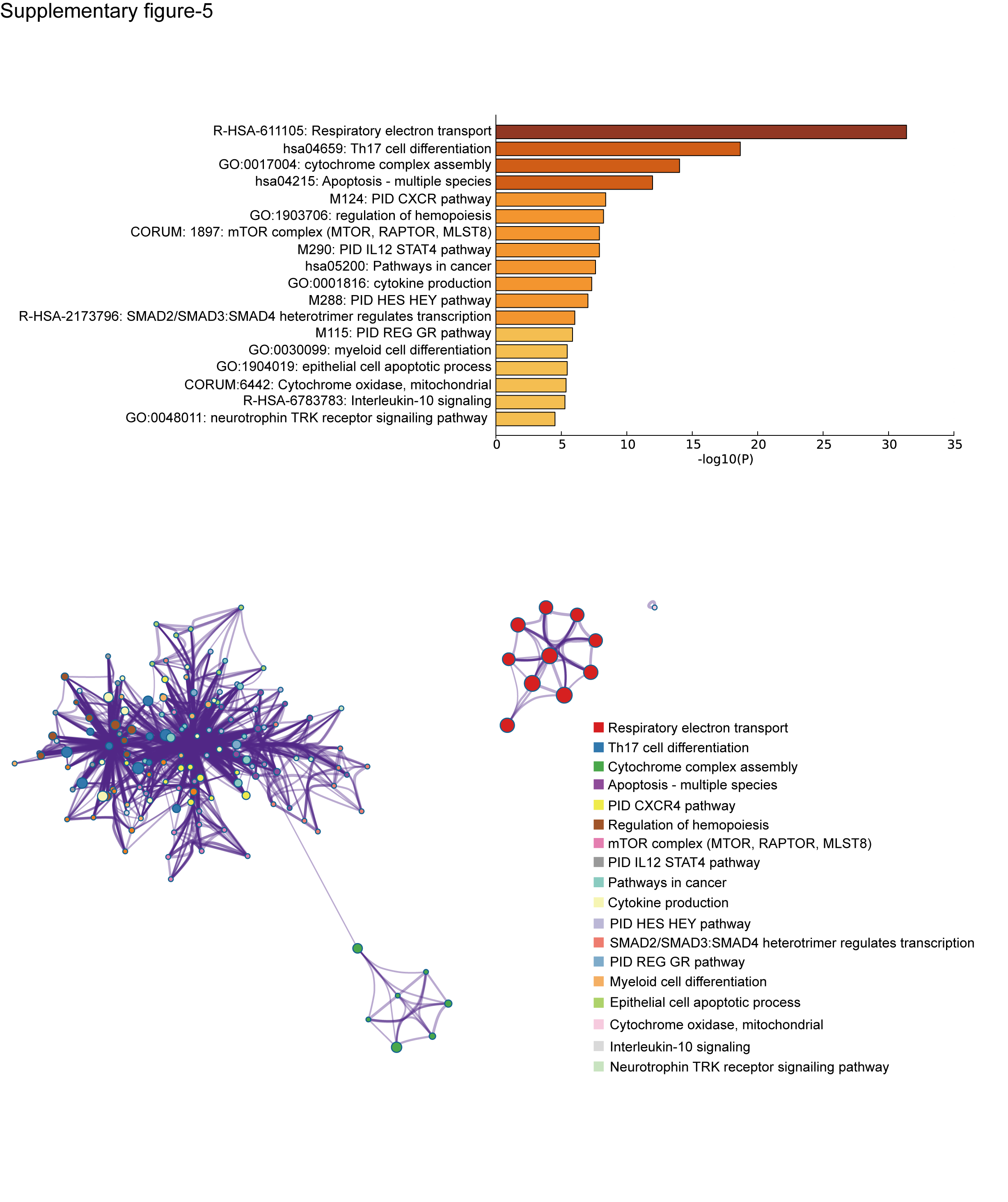

Supplement: Supplementary file 5 [file CAM4-8-593-s005.tif]
